# Characteristics of Gut Microbiota in Patients With Clear Cell Renal Cell Carcinoma

**DOI:** 10.3389/fmicb.2022.913718

**Published:** 2022-07-04

**Authors:** Yang Chen, Junjie Ma, Yunze Dong, Ziyu Yang, Na Zhao, Qian Liu, Wei Zhai, Junhua Zheng

**Affiliations:** ^1^Department of Urology, Renji Hospital, Shanghai Jiao Tong University School of Medicine, Shanghai, China; ^2^Department of Urology, Shanghai Tenth People’s Hospital, School of Medicine in Tongji University, Shanghai, China; ^3^Department of Laboratory Medicine, Renji Hospital, Shanghai Jiao Tong University School of Medicine, Shanghai, China

**Keywords:** gut microbiota, clear cell renal cell carcinoma, *Streptococcus lutetiensis*, proliferation, migration, invasion

## Abstract

Different gut microbiota is implicated in different diseases, including cancer. However, gut microbiota differences between individuals with clear cell renal cell carcinoma (ccRCC) and healthy individuals are unclear. Here, we analyzed gut microbiota composition in 51 ccRCC patients and 40 healthy controls using 16S rRNA sequencing analysis. We observed that *Blautia*, *Streptococcus*, *[Ruminococcus]_torques_group*, *Romboutsia*, and *[Eubacterium]_hallii_group* were dominant and positively associated with ccRCC. We isolated and cultured *Streptococcus lutetiensis* to characterize specific gut microbiota that promotes ccRCC and found that it promoted *in vitro* ccRCC proliferation, migration, and invasion *via* the TGF-signaling pathway. Interactions identified between the gut microbiota and ccRCC suggest the gut microbiota could serve as a potential non-invasive tool for predicting ccRCC risk and also function as a cancer therapy target.

## Introduction

Globally, renal cell carcinoma (RCC) is the seventh most prevalent cancer (Padala et al., 2020). According to 2020 GLOBOCAN data produced by the International Agency for Research on Cancer (IARC), 431,288 new RCC cases occurred, constituting 2.2% of all cancer diagnoses, with the deaths of 179,368 individuals, and accounting for 1.8% of global cancer deaths. Clear cell renal cell carcinoma (ccRCC) accounts for 70% of RCC and is the most common histological subtype (Kabaria et al., 2016). Approximately two-thirds of RCC cases are localized, but 30–40% tend to progress to metastasis, regardless of complete surgical resection or not (Kim et al., 2017). The 5-year relative survival rate for early renal cancer, characterized as localized tumors in the kidney, is 92% (Kabaria et al., 2016). Although some prognostic markers, such as BAP1 and PBRM1 may predict clinical RCC outcomes (Boorjian, 2014), no definitive markers are yet available to predict the risk of developing RCC.

The gut microbiota is involved in the development of cancer. For example, gut microbiota is correlated with chemo-radiotherapy response rates in colorectal cancer (CRC) (Yi et al., 2021). Moreover, they also impact immunotherapy responses during metastatic RCC (Derosa et al., 2020), which is distant from the gastrointestinal tract. The kidneys interact with the gut microbiota and reflect the “gut-kidney axis” (Giordano et al., 2021). Renal toxins such as trimethylamine oxide (TMAO) originate from microbial metabolites and promote progressive renal tubular-interstitial fibrosis and kidney impairment (Evenepoel et al., 2017). A fecal microbiota transplantation study showed that mice receiving *Eggerthella lenta* and *Fusobacterium nucleatum* exhibited more severe renal fibrosis, glomerulosclerosis and oxidative stress, and increased serum levels of uremic toxins, including p-cresol sulfate, phenylacetylglycine, phenyl sulfate, and indoxyl sulfate, while *Bifidobacterium animalis* decreased the abundance of the aforementioned species and relieving renal impairment severity (Wang et al., 2020). Another study (Denburg et al., 2020), on calcium oxalate kidney stone disease in children showed that oxalate-degrading bacterial taxa, including *E. lenta*, were decreased in children who had kidney stones, and suggested gut microbiota dysbiosis caused this disease by perturbing the “gut-kidney axis.”

Herein, we investigated differences in gut microbiota diversity between ccRCC and healthy controls (HCs) and identified five biomarkers in a model predicting the ccRCC incidence rate. In addition, we shed light on how gut microbiota affects ccRCC development, which could be used as a potential target to inhibit ccRCC.

## Materials and Methods

### Patients

In total, 51 ccRCC patients and 40 HCs at Renji Hospital, Shanghai Jiao Tong University School of Medicine, were recruited between March 2021 and June 2021. ccRCC patients were diagnosed according to histology. Inclusion criteria include (1) normal blood glucose and blood pressure, (2) normal liver and kidney function test ranges, and (3) not taking antibiotics or prebiotics three months before fecal sample collection. Fecal samples were collected according to the approved protocol by the Renji hospital Ethics Committees ahead of the procedure of the enrollment, and written informed consent was obtained from both groups. All fecal samples were freshly collected and frozen at −80°C.

### DNA Extraction, 16S rRNA Gene Amplicon Sequencing, and Data Processing

Total genomic DNA from fecal samples was extracted using the acetyl trimethylammonium bromide (CTAB) method (Bailey et al., 2022). DNA concentration and purity were monitored using 1% agarose gels. The 16S rRNA V3–V4 region was amplified and sequenced using NovaSeq PE250 (Illumina, CA, United States). Sequencing libraries were generated using the TruSeq^®^ DNA PCR-Free Sample Preparation Kit (Illumina, CA, United States) and index codes were added to the following manufacturer’s recommendations. Library quality was assessed using the Qubit@ 2.0 Fluorometer (Thermo Scientific, MA, United States) and the Agilent Bioanalyzer 2100 system (Agilent Technologies Inc., CA, United States). The library was then sequenced on an Illumina NovaSeq platform, with 250 base pair (bp) paired-end reads generated and assigned to each sample based on barcodes and then merged with FLASH (V1.2.7) (Magoč and Salzberg, 2011). Quality filtering of raw tags was performed under specific filtering conditions to generate high-quality clean tags (Bokulich et al., 2013) according to Quantitative Insights Into Microbial Ecology (QIIME, V1.9.1) (Caporaso et al., 2010) quality control processes. Tags were compared with a reference database (Silva) using the UCHIME algorithm (Edgar et al., 2011) to detect and remove chimeric sequences (Haas et al., 2011), generating effective tags.

Sequence analysis was performed in Uparse software (v7.0.1001) (Edgar, 2013). Sequences with ≥97% similarity were assigned to the same operating taxonomic units (OTUs). For each representative sequence, the Silva database (Quast et al., 2013) was used based on Mothur algorithms to annotate taxonomic information. To study the phylogenetic relationships between different OTUs, and differences in dominant species between different samples (groups), multiple sequence alignments were conducted using MUSCLE software (Version 3.8.31^1^) (Edgar, 2004). OTU abundance was normalized using a standard sequence number corresponding to the sample with the least sequences. We calculated α- and β-diversity in QIIME (Version 1.7.0 and 1.9.1, respectively) and displayed data using R version 4.1.1 (R Foundation for Statistical Computing, Vienna, Austria) and GraphPad Prism 6 software (GraphPad Software, Inc., CA, United States). Tax4Fun function predictions were processed using the nearest neighbor method based on minimum 16S rRNA sequence similarity (Aßhauer et al., 2015).

### Bacterial Strains and Culture Conditions

*Streptococcus lutetiensis* was derived from ccRCC fecal samples and grown at 37°c in brain-heart infusion (BHI) broth, or on Columbia blood agar plates (BKMAM, China). Starter cultures were prepared by growing strains overnight in 3 ml BHI broth. The overnight culture was diluted at 1:100 in fresh BHI broth. Cells were harvested at the exponential growth phase; optical density (OD) = 0.8 at 600 nm, measured by a microplate reader (BioTek, VT, United States).

### Cell Lines

Human RCC ACHN and A498 cells were cultured in Modified Eagle Medium (MEM) (Servicebio, China) containing 10% fetal bovine serum (FBS) (Thermo Scientific, MA, United States) and 1% penicillin/streptomycin (Servicebio, China) at 37°C with 5% CO_2_. Cell lines were obtained from the American Type Culture Collection (Manassas, VA, United States).

### 5-Ethynyl-2′-Deoxyuridine Assay

An 5-ethynyl-2′-deoxyuridine (EdU) incorporation assay was performed using the Cell-Light™ EdU Apollo^®^567 *in vitro* flow cytometry kit (Ribobio, China) according to the manufacturer’s instructions. Briefly, A498 and ACHN cells were cultured in MEM supplemented with 50 μM EdU for 2 h at 37°C and washed three times in cold phosphate-buffered saline (PBS) (Servicebio, China). Cells were fixed in 4% paraformaldehyde (Servicebio, China), resuspended in 100 μl 1 × Apollo reaction buffer (Ribobio, China), and incubated at room temperature for 30 min. Cells were then washed twice in PBS plus 0.5% Triton X-100 (Thermo Scientific, MA, United States), stained in 1 × Hoechst 33342 reaction buffer (Ribobio, China) for 30 min at room temperature, washed twice in PBS, and finally resuspended in 300 μl PBS. Cells were observed using an inverted immunofluorescence microscope at ×10 magnification (Olympus, Japan).

### Wound Healing Assay

Upon reaching 90% confluence, cell monolayers were scratched with a 200 μl sterile pipette tip, with detached cells washed away in PBS. Then, cells were cultured at 37°C in MEM plus 2% FBS. Cells migrating into wound sites were analyzed over a 20 h period, with plates imaged at 0 and 20 h using an inverted light microscope (magnification ×100, Olympus). Cell migration was quantified by determining the extent (%) of wound healing using ImageJ (v1.52a) (Wayne Rasband, United States); (0 h scratch area − 24 h scratch area)/0 h scratch area × 100%.

### Transwell Invasion Assay

Cell invasion was analyzed using Transwell chambers pre-coated with 20% Matrigel (Corning, NY, United States) for 20 h at 37°C. Briefly, 5 × 10^4^ cells in serum-free MEM were added to the upper chambers of Transwell inserts, while MEM plus 10% FBS (chemoattractant) was added to the lower chambers. Following incubation for 20 h at 37°C, non-invaded cells were removed, and invading cells in the lower chambers were fixed in absolute ethanol for 10 min and stained in 0.5% crystal violet (Servicebio, China) for 10 min at room temperature. Invaded cells were counted in one randomly selected field from each chamber using an inverted light microscope (magnification ×100; Olympus).

### Quantitative Real-Time PCR

RNA was extracted using QIAzol lysis reagent (Qiagen, Germany) according to the manufacturer’s instructions. RNA concentrations were measured and samples were stored at −80°C. Reverse transcription was performed according to instructions from the 4 × Reverse Transcription Master Mix kit (EZBioscience, MN, United States).

*Streptococcus lutetiensis* DNA levels were determined using quantitative real-time PCR (qRT-PCR), which targeted the single-copy gene, superoxide dismutase A (*sodA*), previously used to distinguish among *Streptococcus* spp. (Alber et al., 2004). Primer sequences were analyzed using the basic local alignment search tool (BLAST)^2^ against the GeneBank nucleotide database to confirm primer specificity toward *S. lutetiensis*. qRT-PCR was performed in a 20 μl reaction volume containing: 2 × Color SYBR Green qPCR Master Mix (Low ROX) (Yeasen, China), 10 μM each primer [*S. lutetiensis sodA* forward primer; (GGTGGTGGTGCTCTTAACCA) and *S. lutetiensis sodA* reverse primer (ACGTGTTGTTGCAGCTTTTGT)], and 2 μl DNA from ccRCC fecal samples. The negative control contained DNA from HC fecal samples. Amplification was performed on the QuantStudio 7 Flex Real-Time PCR system (ABI, MA, United States) using the following cycling parameters: denaturation at 95°C for 5 min, and 40 cycles of 95°C for 10 s and 60°C for 30 s. Bacterial loads were determined using standard curves. Samples and standard curves were run in triplicate. Samples with cycle threshold values ≤36 were considered positive for *S. lutetiensis*.

### Western Blotting

Cells were cultured in an appropriate medium in the presence of *S. lutetiensis* conditioned medium (Sl.CM) or BHI for 6 h and then washed three times in sterile PBS. Cells were then lysed in T-PER Tissue Protein Extraction Reagent (Thermo Scientific, MA, United States), lysates were centrifuged at 12,000 rpm for 10 min, and quantified proteins underwent sodium dodecyl sulfate-gel polyacrylamide gel electrophoresis and Western blotting. The following primary antibodies were used: rabbit GAPDH (1:1,000, Cell Signaling Technology, MA, United States), TGF-β2 (1:1,000, Proteintech, PA, United States), and SMAD2 (1:1,000, Cell Signaling Technology, MA, United States). Membranes were rinsed three times in TBST (Servicebio, China) for 10 min each. Then, membranes were incubated with secondary horse radish peroxidase-conjugated Affinipure Goat Anti-Rabbit IgG antibody (H + L) (1:5,000, Proteintech, PA, United States). Membranes were immersed in super enhanced chemiluminescent plus reagent (Advansta, CA, United States) to develop luminescence.

### Statistical Analysis

Significant differences in clinical characteristics were evaluated using an unpaired *t*-test or Fisher’s exact test. Differences were significant when *p* < 0.05. The two-tailed Student’s *t*-test was performed and the *p*-value adjusted by the Benjamini–Hochberg (BH) correction. The probability level for statistical analysis was set at α = 0.05 and was adjusted by the BH correction to allow for a maximum 5% probability (*q* = 0.05) of a false positive detection. All data were analyzed in Graph Pad Prism 6 software, R version 4.1.1, and Microsoft Excel (Microsoft Corporation, Seattle, WA, United States).

## Results

### Clinical Characteristics of Clear Cell Renal Cell Carcinoma Patients and Healthy Controls

**FIGURE 1:**
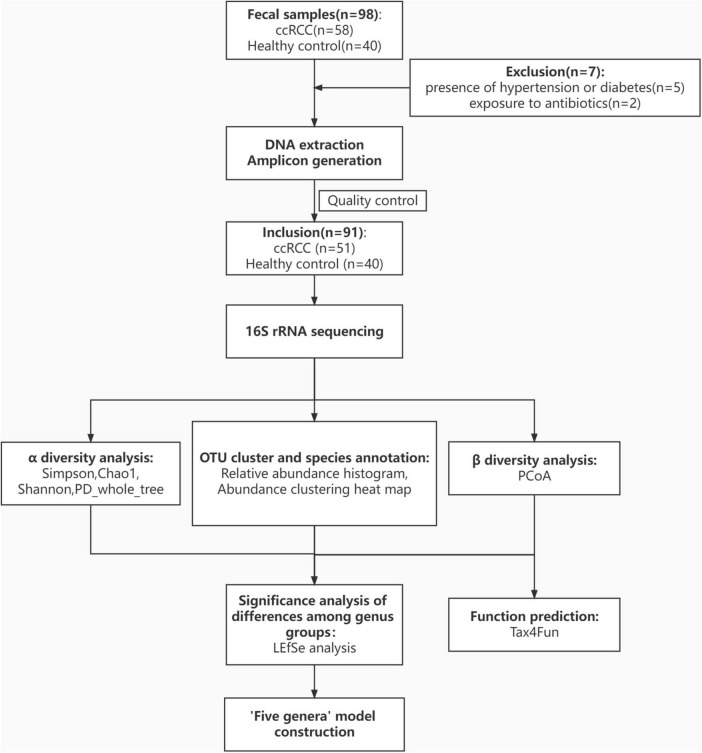
Study design and flow diagram. In total, 98 fecal samples from Renji Hospital were prospectively collected. After excluding 5 patients for the presence of hypertension or diabetes and 2 patients for exposure to antibiotics, 51 patients with ccRCC, and 40 healthy controls were included and their gut microbiota was characterized. ccRCC, clear cell renal cell carcinoma; HC, healthy control.

All ccRCC patients and HCs were enrolled at Renji Hospital. In total, 98 fecal samples were prospectively collected. After a strict screening process, 51 ccRCC patients and 40 HCs were included for 16S rRNA gene amplicon sequencing (Figure 1). Patient demographics and clinical characteristics are collected from hospital electronic medical records. Clinicopathological variables [gender, age, body mass index (BMI), and tumor-node-metastasis (TNM) stage] (Table 1) from both groups were generally matched.

**TABLE 1:** Clinical characteristics of ccRCC patients and healthy controls.

	ccRCC (*n* = 51)	HC (*n* = 40)	*p*-value
Age (years) (mean ± SD)	45.69 ± 11.58	45.50 ± 8.038	0.931
BMI (mean ± SD)	24.18 ± 4.11	24.31 ± 2.86	0.865
**Gender**			
Male	35 (61.4%)	22 (38.6%)	0.182
Female	16 (47.1%)	18 (52.9%)	
**T stage**			
T1a	43 (84.31%)	N/A	
T1b	8 (15.69%)		
**ccRCC grade**			
1	13 (25.49%)	N/A	
2	30 (58.82%)		
3	8 (15.69%)		

*BMI, body mass index; T stage, tumor stage; ccRCC, clear cell renal cell carcinoma; SD, standard deviation; N/A, not applicable. Group clinicopathological variables (age, BMI, and gender) were generally matched. Unpaired t-test was used to compare age and BMI between ccRCC patients and healthy controls; Fisher’s exact test was used to compare gender distribution between the two groups.*

### Increased Gut Microbial Diversity and Microbial Function in Clear Cell Renal Cell Carcinoma Patients

**FIGURE 2:**
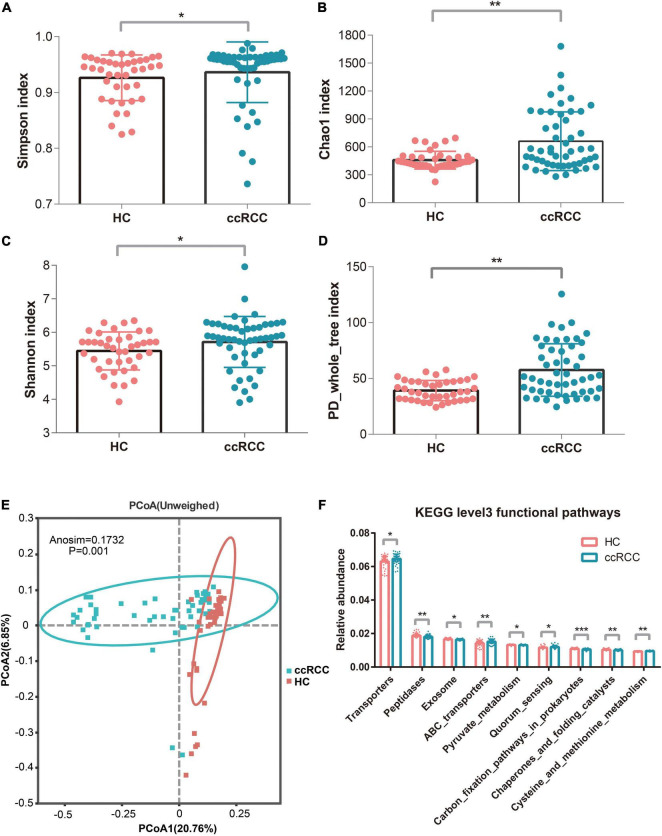
Increased gut microbiota diversity and microbial functions in ccRCC group (*n* = 51) vs. HC group (*n* = 40). Alpha diversity, estimated by Simpson **(A)**, Chao1 **(B)**, Shannon **(C)**, and PD_whole_tree index **(D)** were significantly up-regulated in ccRCC group when compared with HC group (*p* = 0.013, 0.0041, 0.014, and 7.68e–05, respectively by Wilcoxon rank-sum test). **(E)** PCoA based on unweighted UniFrac values showed a different taxonomic composition between the ccRCC and HC groups. **(F)** Tax4Fun analysis identified significant differences in microbial genes between the two groups. Results are presented as the mean ± SD. Statistical evaluations were performed using the Kruskal–Wallis test followed by Dunn’s multiple comparisons test. *p < 0.05, **p < 0.01, *** *p* < 0.001. ccRCC, clear cell renal cell carcinoma; HC, healthy control; PCoA, principal coordinates analysis; SD, standard deviation.

To evaluate differences in bacterial diversity between groups, sequences were aligned to estimate α- and β-diversity. An increased α-diversity was observed in ccRCC group (*n* = 51) vs. HC group (*n* = 40), as measured by Simpson, Chao1, Shannon, and PD_whole_tree indices (*p* = 0.013, 0.0041, 0.014, and 7.68e−05, respectively) using the Wilcoxon rank-sum test) (Figures 2A–D). To indicate microbiome space between groups, β-diversity was calculated using principal coordinate analysis (PCoA) based on unweighted UniFrac values to evaluate variations in community composition. When compared to the HC group, the ccRCC group had a significantly different overall microbiome structure [analysis of similarities (ANOSIM) = 0.1731 and *p* = 0.001] (Figure 2E). When we examined differentially abundant microbial genes using Tax4Fun, significant differences were identified between groups. These genes involved in transporters, quorum sensing, and cysteine and methionine metabolism were significantly more active in the ccRCC group (Figure 2F).

### Characteristic Gut Microbiota in Clear Cell Renal Cell Carcinoma Group

**FIGURE 3:**
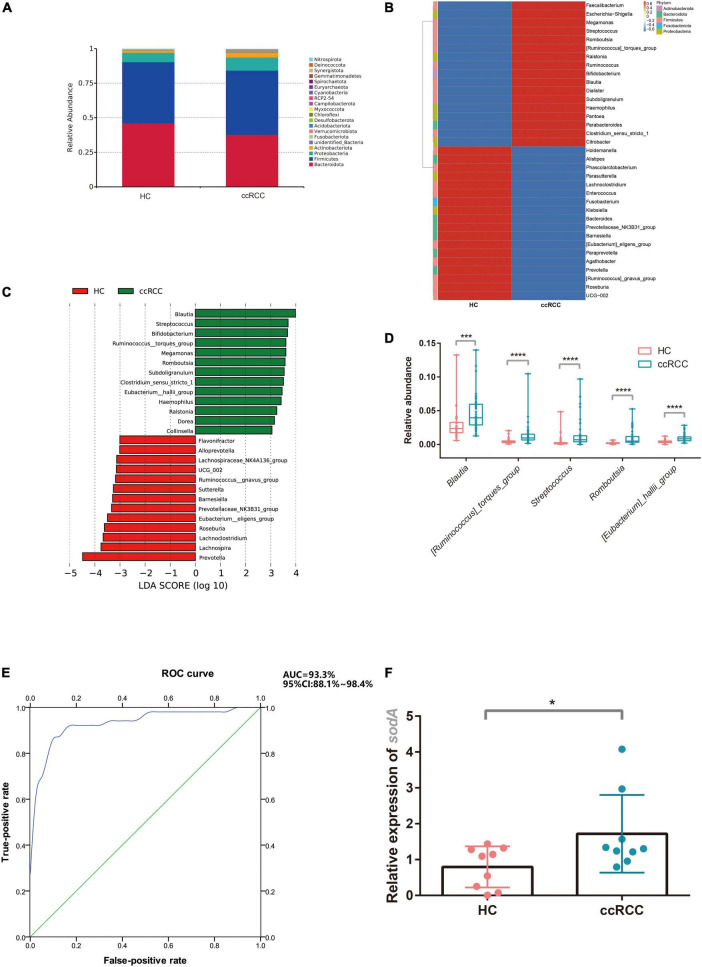
Characteristic gut microbiota in ccRCC and HC groups. The gut microbiota composition at phylum **(A)** and genus levels **(B)** between groups was analyzed. **(C)** LEfSe analysis indicated group-associated microbiota at the genus level. Bars on the right of zero represent microbiota enriched in the ccRCC group, whereas bars left of zero represent microbiota enriched in the HC group (LDA > 3). **(D)** Five representative genera “*Blautia, Streptococcus, [Ruminococcus]_torques_group, Romboutsia, [Eubacterium]_hallii_group*” were analyzed **(E)** to build a receiver operating characteristic curve of a “five-genera” model predicting RCC risk. **(F)** *S. lutetiensis* levels were determined using qPCR targeting *sodA*. Results are presented as the mean ± SD. Statistical evaluations were performed using the Kruskal–Wallis test followed by Dunn’s multiple comparisons test. *p < 0.05, ***p < 0.001, ****p < 0.0001. ccRCC, clear cell renal cell carcinoma; HC, healthy control; LEfSe, linear discriminant analysis effect size; LDA, linear discriminant analysis; PCoA, principal coordinates analysis; *sodA*, superoxide dismutase A; SD, standard deviation.

Gut microbiota composition in the ccRCC group at the phylum level was shown (Figure 3A). On average, the combined bacterial phyla of *Firmicutes*, *Bacteroidetes*, and *Proteobacteria* accounted for more than 80% of the sequences and were dominant populations in both groups. At the genus level, the top 35 genera, including *Bacteroides*, *Faecalibacterium*, *Prevotella*, and *Escherichia-Shigella* are shown (Figure 3B). To compare differences in gut microbiota between groups, linear discriminant analysis effect-size (LEfSe) was used to identify specific bacteria associated with ccRCC (Figure 3C). The five genera, *Blautia, Streptococcus, [Ruminococcus]_torques_group, Romboutsia*, and *[Eubacterium]_hallii_group* were all significantly overrepresented [all linear discriminant analysis (LDA) scores (log10) > 3] in fecal samples from ccRCC group, whereas *Prevotella, Lachnospira, Lachnoclostridium*, and *Roseburia* were the most abundant microbiota in the HC group [LDA scores (log10) > 3]. To illustrate the predictive value of gut microbiota for ccRCC, we selected five genera positively associated with ccRCC from LEfSe analyses: *Blautia, Streptococcus, [Ruminococcus]_torques_group, Romboutsia*, and *[Eubacterium]_hallii_group* (Figure 3D). This “five-genera” analysis accurately predicted ccRCC as demonstrated by a receiver operating characteristic curve (AUC = 93.3%) and suggested predictive models based on genera could serve as biomarkers for ccRCC (Figure 3E).

To test whether the “five-genera” model indeed contributed to ccRCC progression, we tried to isolate the bacteria from ccRCC fecal samples. Unfortunately, we failed to isolate other strains among the “five-genera” from ccRCC fecal samples except for *S. lutetiensis*. The abundance of *S. lutetiensis* was also higher in the ccRCC group when compared with the HC group by testing the expression of *sodA* from fecal samples (Figure 3F), we hypothesized that *S. lutetiensis* contributes to the progression of ccRCC. So *S. lutetiensis* was used for the following experiment.

### *Streptococcus lutetiensis* Conditioned Medium Promotes Clear Cell Renal Cell Carcinoma Cell Proliferation, Migration, and Invasion

**FIGURE 4:**
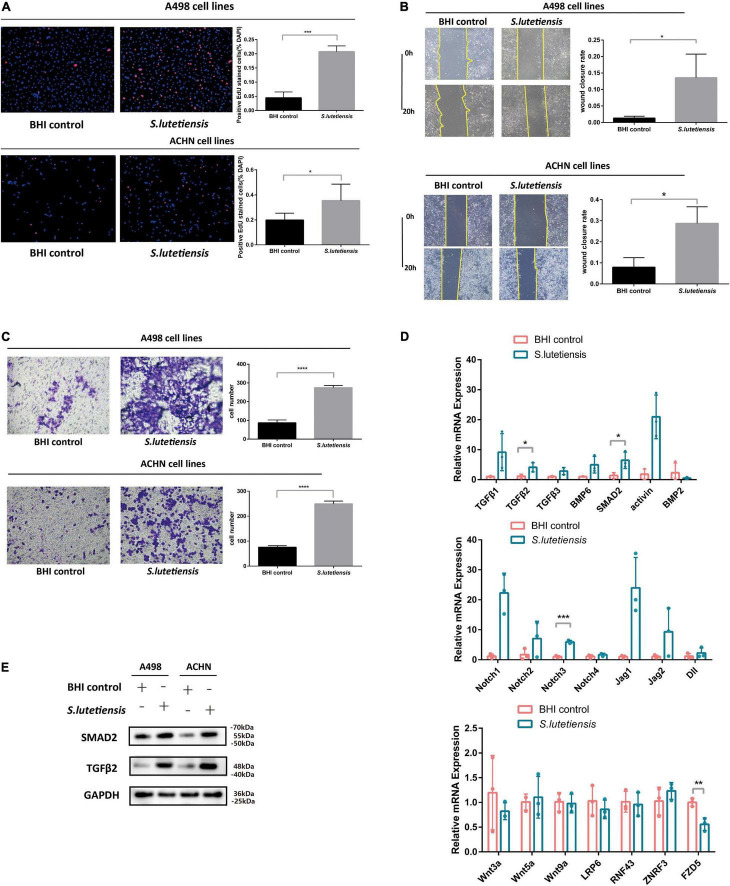
*Streptococcus lutetiensis* conditioned medium (Sl.CM) promotes A498 and ACHN cell proliferation, migration, and invasion through the *TGF*-β signaling pathway. **(A)** EdU assay showed that Sl.CM increased A498 and ACHN cell proliferation. **(B)** Wound healing assay showed that Sl.CM increased A498 and ACHN cell migration. **(C)** Transwell invasion assay showed that Sl.CM increased A498 and ACHN cell invasion. **(D)** qRT-PCR showed that mRNA expression of *TGF-β2* and *SMAD2* in *TGF*-β signaling pathway was increased in Sl.CM treated ACHN cells but mRNA expression of *Wnt* and *Notch* signaling pathway showed no obvious difference between the two groups. **(E)** Western blot showed that *TGF-β2* and *SMAD2* protein expression were increased in Sl.CM treated A498 and ACHN cells. Results are presented as the mean ± SD and statistical significance was determined using the unpaired *t*-test. *p < 0.05, **p < 0.01, ***p < 0.001, ****p < 0.0001. Sl.CM, *S. lutetiensis* conditioning medium; EdU, 5-ethynyl-2′-deoxyuridine; SD, standard deviation.

To determine if *S. lutetiensis* promoted ccRCC *in vitro*, the RCC cell lines, A498 and ACHN, were co-cultured with or without Sl.CM. BHI was used as a blank control. *S. lutetiensis* was cultured overnight in a BHI medium, bacteria were removed by centrifugation, and the supernatant was filtered through a 0.22-μm membrane to generate the Sl.CM, which was used to treat A498 and ACHN cells at 12.5% (vol/vol) for 6 h. An EdU assay was performed to determine the effect of Sl.CM on cell proliferation. The results demonstrated that Sl.CM increased A498 and ACHN cell proliferation. As shown in Figure 4A, the percentage of EdU-positive cells was significantly higher in the *S. lutetiensis* group (0.21 ± 0.02) when compared to BHI control (0.04 ± 0.02) (*p* < 0.001) in A498 cell line. A similar result could also be obtained in the ACHN cell line as the number of EdU-positive cells was higher in *S. lutetiensis* group (0.35 ± 0.13) when compared to the BHI control (0.20 ± 0.06) (*p* < 0.05). A wound healing assay was performed to determine the effect of Sl.CM on A498 and ACHN cell migration. The results demonstrated that Sl.CM increased A498 and ACHN cell migration. As shown in Figure 4B, the wound closure rate was higher in *S. lutetiensis* group (0.14 ± 0.07) when compared to BHI control (0.01 ± 0.01) (*p* < 0.05) in the A498 cell line. A similar result could also be obtained in the ACHN cell line as the wound closure rate was significantly higher in *S. lutetiensis* group (0.29 ± 0.08) when compared to the BHI control (0.08 ± 0.05) (*p* < 0.05). Transwell invasion assay was performed to determine the effect of Sl.CM on A498 and ACHN cell invasion. The results demonstrated that Sl.CM increased A498 and ACHN cell invasion. As shown in Figure 4C, the number of transmembrane cells was significantly higher in *S. lutetiensis* group (274.30 ± 11.85) when compared to BHI control (86.67 ± 15.37) (*p* < 0.0001) in the A498 cell line. A similar result could also be obtained in the ACHN cell line as the number of transmembrane cells was significantly higher in *S. lutetiensis* group (249.00 ± 11.53) when compared to BHI control (75.67 ± 6.03) (*p* < 0.0001).

### *Streptococcus lutetiensis* Conditioned Medium Promotes Clear Cell Renal Cell Carcinoma Progression Through *TGF*-β Signaling Pathway

These observations suggest *S. lutetiensis* promoted ccRCC development, but the underlying mechanisms were unclear. Previous studies reported that some *Streptococcus* species activated *TGF*-β signaling in various diseases, e.g., *Streptococcus pneumoniae* induced *TGF*-β1 expression during tympanosclerosis pathogenesis (Qiu et al., 2021), asthma inflammation (Schmit et al., 2020) and pulmonary infection (Peteranderl et al., 2017). So we theorized that *S. lutetiensis* promoted ccRCC *via TGF*-β signaling, to explore this possibility, the ACHN cell line was co-cultured with Sl.CM or BHI and the mRNA expression levels of the *TGF*-β signaling pathway were assessed by qRT-PCR. We also assessed the mRNA expression levels of *Wnt* and *Notch* signaling pathways. As shown in Figure 4D, *S. lutetiensis* increased relative expression levels of the *TGF-β2* (4.11 ± 1.52) (*p* < 0.05) and *SMAD2* (6.45 ± 2.73) (*p* < 0.05) genes in *TGF*-β signaling pathway while upregulating little markers in the *Wnt* and *Notch* signaling pathway. Then we investigated related protein expression levels in A498 and ACHN cell lines. As shown in Figure 4E, *TGF-β2* and *SMAD2* protein levels in Sl.CM-co cultured RCC cell lines were significantly increased when compared with BHI control, thus verifying our hypothesis.

## Discussion

In this study, we investigated gut microbiota specific to ccRCC and successfully constructed a prediction model for disease risk based on five biomarkers with high AUC. We also showed that gut microbiota affected ccRCC progression. Using gut microbiota as a diagnostic tool for tumorigenesis has gained considerable traction in research. In China, a large clinical cohort study involving 419 fecal samples reported that gut microbiota such as *Klebsiella* and *Haemophilus* could be used as potential non-invasive tools for early HCC diagnosis (Ren et al., 2019). In colon cancer, 17 CRC-specific metabolites were identified, with polyamines such as cadaverine and putrescine strongly correlated with CRC (Yang et al., 2019). Surprisingly, in cancers distant from the gut, the gut microbiota also accurately predicted cancer. A predictive model of 13 OTU-based markers, including *Veillonella* and *Ruminococcus*, accurately predicted lung cancer (Zheng et al., 2020). Also, *Bacteroides* and *Streptococcus* in prostate cancer patients were more abundant, with PICRUSt data showing that B vitamin metabolic pathways were down-regulated in these patients, potentially indicating protective effects from prostate cancer risk (Liss et al., 2018), whereas in other work, *Prevotella stercorea* provided an alternative source of androgen and contributed to endocrine resistance in castration-resistant prostate cancer (Pernigoni et al., 2021).

In RCC, recent studies reported that the oral administration of commensal bacteria, such as *Akkermansia muciniphila*, was enriched in patients experiencing immunotherapy clinical benefits (Salgia et al., 2020) while *Clostridium hathewayi* was enriched in non-response groups and related to recent antibiotic-use in metastatic RCC patients. A randomized clinical trial involving metastatic RCC patients with TKI-related diarrhea showed that fecal material from healthy donors greatly relieved their symptoms, while *A. muciniphila* was shared between healthy donors and patients with improved outcomes after receiving feces from healthy donors (Ianiro et al., 2020).

From our study, *Blautia*, *[Ruminococcus]_torques_group*, *Streptococcus*, *Romboutsia*, and *[Eubacterium]_hallii_group* genera were enriched in the ccRCC group. *Blautia* (Deng et al., 2021), *Romboutsia* (Deng et al., 2021), and *Streptococcus* (Deng et al., 2021; Jiang et al., 2021) were reportedly enriched in esophageal cancer (Jiang et al., 2021). *Streptococcus* was also abundant in CRC (Yang et al., 2021). *Streptococcus* is a Gram-positive bacteria in humans and is mostly associated with urinary tract infection, sepsis, endocarditis, and CRC. Some studies have shown that *Streptococcus* induced inflammation and carcinogenesis by activating COX-2 which induced angiogenesis and suppressed apoptosis (Dalal et al., 2021), activating interleukin-8 (IL-8) which is the production of angiogenic factors (Dalal et al., 2021), or activating NF-κB which is a transcriptional factor involved in inflammation (Abdulamir et al., 2009). *Streptococcus* infection could activate the NLRP3 inflammasome and induce IL-1β secretion (Harder et al., 2009), which was previously shown to promote colon cancer (Wang et al., 2021). *Streptococcus* was also more abundant in prostate cancer, while PICRUSt data indicated that B vitamin metabolic pathways, which are protective against prostate cancer risk, were down-regulated in prostate cancer patients (Liss et al., 2018). Surprisingly, *Streptococcus* was more abundant in ccRCC tissue than in normal kidney tissue, which suggested an association with tumorigenesis (Liss et al., 2020). In our study, using Tax4Fun functional predictions, ABC transporter genes were more active in ccRCC. Several studies reported that the ABC transporters, ABCB1 (Watanabe et al., 2017) and ABCG2 (Watanabe et al., 2017; Reustle et al., 2018) were correlated with drug resistance in ccRCC, while ABCG2 (Low et al., 2016) was correlated with drug-induced toxicity. ABCB1 expression may also contribute to the side population cell isolation from the 769P cell line and serve as a mediator in the cancer stem cell pathogenesis of ccRCC (Huang et al., 2016). ABCB8 was also reported as a risk predictor for worse clinical outcomes in ccRCC (Blajan et al., 2021). Our data indicated that quorum sensing was more active in ccRCC. This process is key for microbial biofilm formation, which regulates bacterial active efflux pumps to effectively discharge drugs from systems, thereby promoting multidrug resistance (Chew et al., 2020). Quorum sensing signaling molecules induced the disorder of intestinal goblet cell structure and function, ultimately destroying the intestinal mucus barrier (Xiao et al., 2022). Similarly, cysteine and methionine metabolic functions were also more active in ccRCC. Cysteine promoted colon cancer growth *in vitro* and *in vivo*, while it conferred resistance to oxaliplatin and irinotecan chemotherapy (Wu et al., 2021). Conversely, pyruvate metabolism was inactive during ccRCC. Down-regulated pyruvate kinase activity contributes to aerobic glycolysis in ovarian cancer cells and disease progression (Sun et al., 2021).

*Streptococcus lutetiensis* is Gram-positive (Poyart et al., 2002) and produces several exotoxins (Chen et al., 2021) which may be pathogenic, e.g., diarrhea (Jin et al., 2013) or neonatal meningitis with empyema (Yu et al., 2021). In our study, *S. lutetiensis* promoted ccRCC *via TGF*-β signaling, whereas other studies suggested *S. lutetiensis* virulence genes were also associated with carcinogenesis. The endotoxin encoding genes, *SpeG* (encoding pyrogenic exotoxin G) and *smeZ* (encoding mitogenic exotoxin Z), were detected in *S. lutetiensis* (Chen et al., 2021). Exotoxins increased the levels of C-C motif chemokine ligand 2 (Emmer et al., 2008), which aided tumor-associated macrophages (TAM) and cancer progression (Liu et al., 2020). TAMs have previously been shown to promote the progression of ccRCC (Gu et al., 2021).

The limitation of 16S rRNA sequencing is that it can only determine microbiota composition at the genus level. More precise detection methods like culture or metagenomic sequencing can be applied to identify the exact strains. Furthermore, multi-center studies are necessary for validation. Our ultimate goal was to explore gut microbiota potentially affecting ccRCC pathogenesis. To that end, we developed a “five-genera” model that could detect ccRCC and suggested that *S. lutetiensis* may promote ccRCC progression *via* the TGF pathway.

## Data Availability Statement

The datasets presented in this study can be found in online repositories. The names of the repository/repositories and accession number(s) can be found below: NCBI BioProject – PRJNA842560.

## Ethics Statement

The studies involving human participants were reviewed and approved by the Renji Hospital Ethics Committee, Shanghai Jiao Tong University School of Medicine. The patients/participants provided their written informed consent to participate in this study. Written informed consent was obtained from the individual(s) for the publication of any potentially identifiable images or data included in this article.

## Author Contributions

YC designed and finalized the study. YC, JM, and YD performed the experiments. NZ and ZY helped on samples collection and the bioinformatics analysis. YC drew the charts and wrote the manuscript which were revised by WZ, QL, and JZ. QL, WZ, and JZ supervised the project. WZ and JZ funded the project. All authors approved the final version submitted.

## Conflict of Interest

The authors declare that the research was conducted in the absence of any commercial or financial relationships that could be construed as a potential conflict of interest.

## Publisher’s Note

All claims expressed in this article are solely those of the authors and do not necessarily represent those of their affiliated organizations, or those of the publisher, the editors and the reviewers. Any product that may be evaluated in this article, or claim that may be made by its manufacturer, is not guaranteed or endorsed by the publisher.

## Footnotes

1
http://www.drive5.com/muscle/

2
http://blast.ncbi.nlm.nih.gov/
