# Supplementary figures and images for: Characteristics of Gut Microbiota in Patients With Clear Cell Renal Cell Carcinoma
Source: Front Microbiol. 2022 Jul 4;13:913718. doi: 10.3389/fmicb.2022.913718 (PMC9295744; doi:10.3389/fmicb.2022.913718)

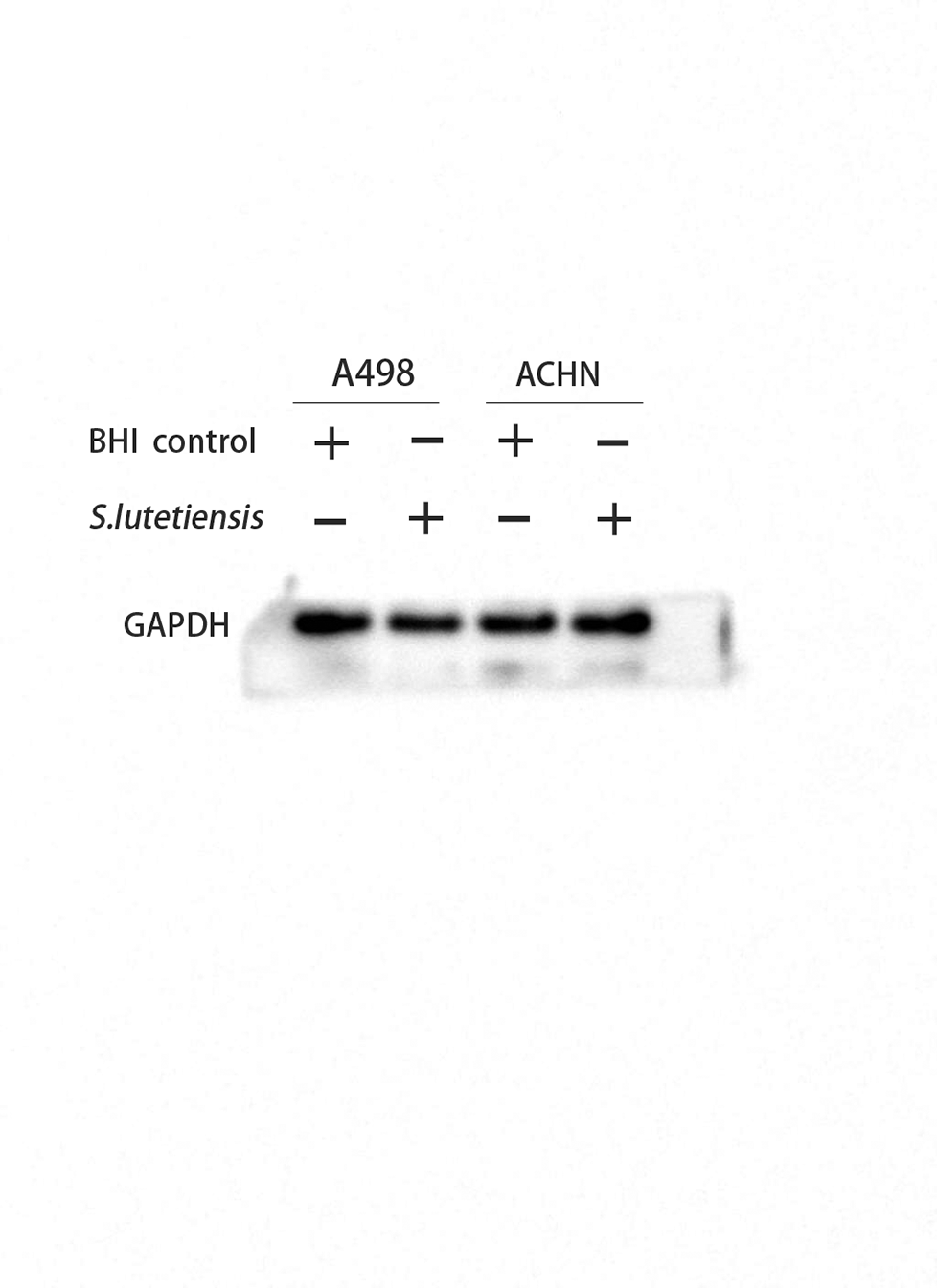

Supplement: Supplementary file 1 [file Image_1.tif]

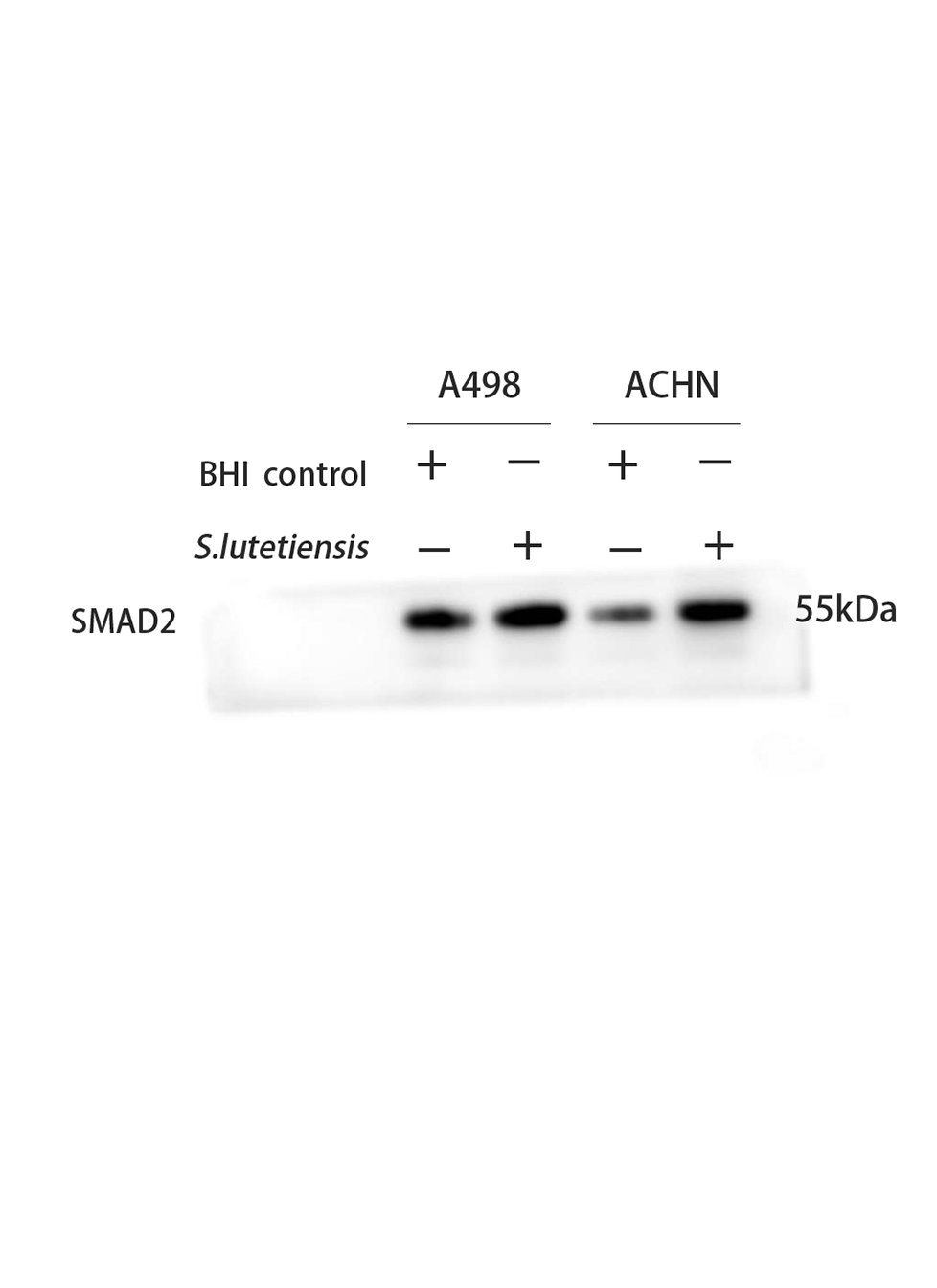

Supplement: Supplementary file 2 [file Image_2.tif]

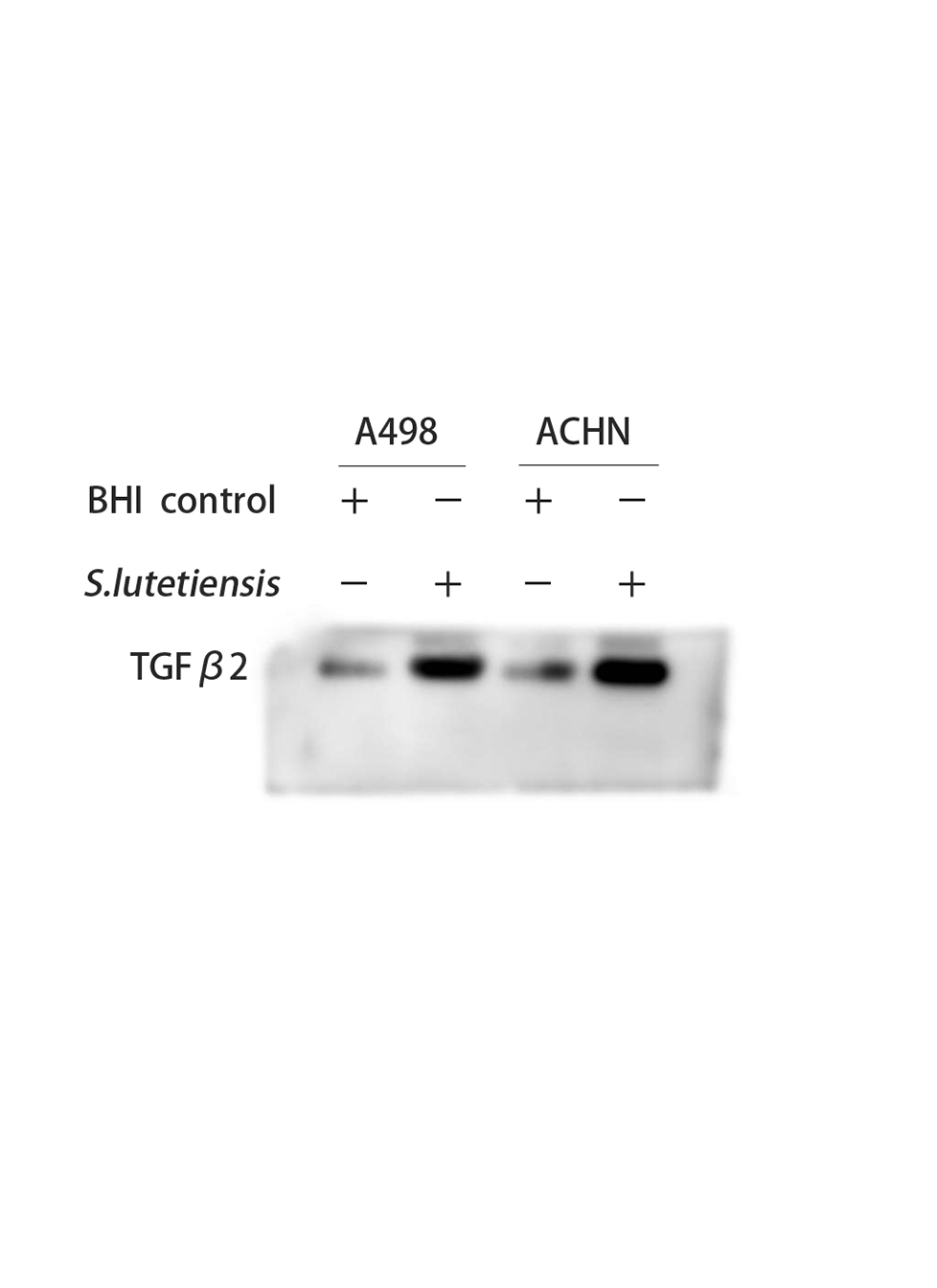

Supplement: Supplementary file 3 [file Image_3.tif]
